# Prophylactic cranial irradiation (PCI) in locally advanced non-small cell lung cancer: time for hope?

**DOI:** 10.18632/oncotarget.25866

**Published:** 2018-08-17

**Authors:** Dirk K. De Ruysscher, Harry J. Groen

**Affiliations:** Maastricht University Medical Center, Department of Radiation Oncology, Maastro Clinic, GROW Research Institute, Maastricht, The Netherlands; Katholieke Universiteit Leuven, Radiation Oncology, Leuven, Belgium

**Keywords:** non-small cell lung cancer, PCI, randomized trial

Brain metastases (BM) occur frequently in lung cancer patients, with an incidence of approximately 30 % in stage IIII non-small cell lung cancer (NSCLC) [1]. When untreated, BM lead to a decreased quality of quality of life (QoL), cause symptoms and are lethal [1]. Whereas historically, BM were treated with corticosteroids to reduce edema and symptoms and whole brain radiotherapy (WBRT), many patients now receive stereotactic radiosurgery (SRS) or systemic therapy [1]. The reason is that WBRT is associated with neuro-cognitive side effects and low intra-cranial tumor control. Nevertheless, all these treatments for established BM are palliative and the overwhelming majority of patients continuously have new BM [2]. Prevention of BM has therefore great potential.

Prophylactic Cranial Irradiation (PCI) was designed for prevention of BM. It is the standard of care for patients with so-called limited stage small cell lung cancer, where it improved the overall survival (OS) at three years, even though the phase III trials were performed without contemporary CT or MRI of the brain and whole body FDG-PET-CT [3]. Most patients in these studies would nowadays been included in studies for metastatic disease. The question is whether this approach also would be favorable to earlier NSCLC.

The recently published phase III study of the NVALT/ DLCRG investigated whether PCI reduces the incidence of symptomatic brain metastases in patients with stage III NSCLC treated with curative intention [4]. All patients were staged with a contrast-enhanced brain CT or MRI and a whole body FDG-PET-CT and were randomized between observation or PCI after concurrent/sequential chemo-radiotherapy with or without surgery. The primary endpoint, the development of symptomatic brain metastases at 24 months, was defined as one or a combination of key symptoms suggesting brain metastases (signs of increased intracranial pressure, headache, nausea and vomiting, cognitive or affective disturbances, seizures and focal neurological symptoms) and MRI or CT proving the existence of brain metastasis. Side effects, survival, quality of life (QoL), quality adjusted survival and health care costs were secondary endpoints. Between 2009 and 2015, 175 patients were randomized, 87 received PCI and 88 observation only. The median follow up was 48.5 months (95% CI, 39–54). The proportion of patients with symptomatic brain metastases was 6/86 (7.0 %) in the PCI group and 24/88 (27.2 %) in the control group (*p* = 0.0005). Moreover, PCI also postponed the time to develop symptomatic brain metastases: HR 0.23 (95% CI 0.09-0.56), *p* = 0.0012). These results are impressive compared to systemic treatments for solid cancers. The median time to develop brain metastases was not reached in either arm. The overall survival was not significantly different between both arms. Grade 1–2 memory impairment (26/86 *vs*. 7/88 and cognitive disturbance (16/86 *vs*. 3/88) were significantly increased in the PCI arm. QoL was only decreased 3 months post-PCI and was similar to the observation arm thereafter. PCI therefore decreases significantly the proportion of patients developing symptomatic brain metastases with an increase of low-grade toxicity.

This recent study confirms other randomized trials that PCI can indeed reduce the proportion of brain metastases, however at the expense of increased grade 1 and 2 toxicity, without showing an effect on the OS [5–9].

As PCI is a highly active treatment, research should continue to optimize it. One of the questions is who are the patients that developed memory and cognitive abnormalities after PCI? Not everybody develops these symptoms. Do they have special characteristics such as more cardiovascular comorbidities?

The neuro-cognitive function could be preserved by sparing the two hippocampus, which are believed to play a major role in the cognitive decline some patients experience [10]. A phase III trial by our group has finished accrual and the results are expected in Q4 of 2018. Pharmacological interventions such as memantine could also decrease neurological side effects. PCI should be investigated together with checkpoint inhibitors and new tyrosine kinase inhibitors (TKI) that cross the blood-brain barrier such as osimertinib,brigatinib, alectinib or lorlatinib. Sequencing of local and systemic treatments and studying the interaction between both modalities will be the next step. Finally, the lack of benefit on OS may be due to a lack of power and a too short follow-up in the published phase III trials. Our group is therefore doing a meta-analysis based on individual, updated patient data of all eligible phase III trials in the world in order to answer the question of PCI lead to an improved OS.

In future, PCI may become standard of care for stage III NSCLC or perhaps in those with ALK translocated or EGFR mutated NSCLC who have prolonged control over their disease. However, more research is needed to study the impact on cognitive integrity and survival.
